# Perceived Stress and Coping Strategies Among Undergraduate Health Science Students of Jimma University Amid the COVID-19 Outbreak: Online Cross-Sectional Survey

**DOI:** 10.3389/fpsyg.2021.639955

**Published:** 2021-03-30

**Authors:** Mengist Awoke, Girma Mamo, Samuel Abdu, Behailu Terefe

**Affiliations:** ^1^Department of Clinical Pharmacy, Faculty of Health Science, School of Pharmacy, Institute of Health, Jimma University, Jimma, Ethiopia; ^2^Faculty of Health Science, School of Nursing, Institute of Health, Jimma University, Jimma, Ethiopia

**Keywords:** perceived level of stress, brief-COPE, associated factors, COVID-19 pandemic, students, Ethiopia

## Abstract

**Background:** The rapid spread of COVID-19 infection has led countries across the globe to take various measures to contain the outbreak, including the closure of Universities. Forcing University students to stay at home has created enormous stress and uncertainty in their daily life.

**Objective:** This study aimed to assess the perceived stress and coping strategies among undergraduate health science students of Jimma University amid the COVID-19 outbreak.

**Materials and methods:** An online cross-sectional survey was conducted involving 337 undergraduate health science students from August to September 5, 2020. The perceived stress scale (PSS)-10 and Brief-COPE scale were used to assess the level of stress and coping strategies, respectively. Statistical Package for Social Science (SPSS) Version 22 was employed for data analysis. Logistic regression was conducted to identify predictors of high perceived stress.

**Results:** The overall mean [±standard deviation (SD)] age of the participants was 22.88 (±1.78) years. The mean (±SD) PSS score was 22.16 (±1.41), and high perceived stress was reported in 121 (35.9%) participants. The overall mean (±SD) coping score was 72.34 (±12.31), and approach coping was the predominantly used strategy for coping with stress. Personal perception of being stressed by the daily number of COVID-19 cases/deaths in Ethiopia (AOR = 4.61, *p* < 0.01), rare online talk/chat with friends (AOR = 4.07, *p* = 0.01), presence of confusion due to the inconsistent strategies developed by the health/government authorities in view of the scientific recommendations (AOR = 2.22, *p* = 0.01), perception of self/family members being at risk of getting sick (AOR = 0.53, *p* = 0.03), decreased household income following the COVID-19 pandemic (AOR = 3.92, *p* = 0.01), practicing denial (AOR = 1.34, *p* < 0.01), self-blame (AOR = 1.23, *p* = 0.02), planning (AOR = 1.28, *p* = 0.01), and religion (AOR = 1.41, *p* < 0.01) as means of coping with stress were associated with high perceived stress.

**Conclusion:** Over one-third of the participants had a high level of perceived stress, and the majority of them were practicing effective means of coping with stress. The authors recommend that the hosting University in collaboration with the concerned bodies develop innovative strategies to improve the psychological well-being of the students.

## Introduction

As of the second half of January 2021, over 98 million COVID-19 infections were recorded globally claiming the lives of over 2.1 million people (Medicine, 2020). In response to the expeditious increase in the number of COVID-19 infections and the World Health Organization‘s declaration of the infection as a pandemic (WHO, 2020b), governments across the world implemented early wide-ranging control measures to mitigate its spread and impact. The measures taken included travel/transportation bans from specific locations, closures of selected institutions and borders, quarantine, self-isolation, and social distancing measures (ACAPS, 2020).

Though the measures adopted to contain the COVID-19 infection were justifiable, the measures greatly impacted the psychosocial, economic, and political aspects of the population across the globe (Bank, 2020; WHO, 2020a). The fear of exposure to infection, subsequent risk of infecting family members and loved ones, prolonged quarantine, fear of relative's death, and concerns about educational progression and other fears have grown tremendously. Thus, COVID-19 has the potential to leave a devastating mark on the psyche of the people, including students across the world (Mohammed et al., 2020; WHO, 2020a).

With the spread of the COVID-19 infection, more than 1.5 billion students and young people across the planet were forced to stay at home due to the closure of schools and Universities, leading to enormous anxiety and uncertainty (Husain, 2020; UNESCO, 2020). Social distancing measures and cessation of face-to-face teaching at higher educational institutes put students in an altogether new position without a well-defined estimate of how long it will last, compromising their daily life (IESALC, 2020). University and college students are especially prone to feelings of loneliness, and they experience higher rates of anxiety and depression compared to the general population (Rahman et al., 2012; Diehl et al., 2018). During the era of COVID-19, due to social isolation, uncertainty, and abrupt transitions, students are prone to further worsening of these feelings (Psychiatry, 2020).

Looking at the experiences of the world, the Ethiopian government took multiple measures, such as closure of schools and restricting large gatherings and movements of people. Additionally, the government adopted WHO's basic measures to reduce transmission of COVID-19 including washing hands frequently using soap, maintaining social distancing, staying informed and following advice given by your healthcare provider, and staying at home if you begin to feel sick. These measures were communicated to the general public through various media platforms (Baye, 2020; Hailu, 2020).

Numerous worldwide studies were conducted to assess the level of perceived stress among University students before the COVID-19 pandemic (Shah et al., 2010; Borjalilu et al., 2015; Ahmed and Prashantha, 2018; Gazzaz et al., 2018; Al-Qahtani and Alsubaie, 2020; Karyotaki et al., 2020; Tariq et al., 2020), but only limited studies were conducted on the same issue during the COVID-19 outbreak. The available studies reported a high perceived stress level for students ranging from 12.6 to 30.2% during the current pandemic (AlAteeq et al., 2020; Pedrozo-Pupo et al., 2020; Sheroun et al., 2020). A study from China revealed that ~25% of the study population experiencing anxiety symptoms, which were positively correlated with increased concerns about academic delays, economic effects of the pandemic, and impacts on daily life (Cao et al., 2020). In Ethiopia, it is estimated that the prevalence of anxiety and depression symptoms has tripled from pre-outbreak estimates (Monitor, 2020). A previous study in Ethiopia revealed that high perceived stress was apparent in more than half of healthcare providers (51.6%) (Chekole et al., 2020), and another study involving University students reported stress in 32.5% of the participants (Aylie et al., 2020).

Medical students experience more stress compared to students of other subjects (Kebede et al., 2019; Olum et al., 2020; Worku et al., 2020). In Ethiopia, age, class, study year, practical attachment, poor/low social support, having stressful life events, financial problems, and thinking about future career expectations were identified as factors related to perceived stress among health science students (Kebede et al., 2019; Worku et al., 2020). On top of pre-existing factors, the unprecedented disruptions in health education and other activities due to the COVID-19 outbreak (Alsoufi et al., 2020) is expected to further affect the mental health of these students. Thus, students had to cope with their own fears, stresses, and insecurity. The way of coping with a stressful event, like the COVID-19 crisis, affects physical health, medical conditions, and emotional well-being either positively or negatively (CDC, 2020). Despite the recent reopening of face-to-face University education, no research has assessed the perceived stress levels and coping strategies of University students during the COVID-19 pandemic in Ethiopia. Therefore, the current study aimed to explore the perceived stress level, associated factors, and stress coping strategies used among health science students of Jimma University amid the COVID 19 outbreak. The hypothesis of this research is that COVID-19-related events increase the risk of high perceived stress level and change stress coping strategies.

## Materials and Methods

### Study Design and Setting

A cross-sectional online surveillance study was conducted at Jimma University from August to September 5, 2020. Jimma University is one of the well-known Universities in Ethiopia, located in the southwest part of the country. It has six colleges and four institutes, one of which is the Institute of Health Sciences (University, 2020), which includes the department of Anesthesia, School of Nursing and Midwifery (Generic Nursing, Generic Midwifery, Operational Theater Nursing, Neonatal Nursing), School of Environmental Health, School of Pharmacy, and School of Laboratory Technology.

### Participants

#### Eligibility Criteria

All undergraduate health science students of Jimma University who were willing and able to respond to online questionnaires were included in the study. A total of 337 health science students participated in the study.

### Variables

#### Independent Variables

Sociodemographic variables (age, sex, residence, living with your parents or family, family size, marital status, field of study, academic year, and household average monthly income), COVID-19-related experience variables, and variables related to measures taken for the prevention of COVID-19.

#### Dependent Variables

Perceived stress level and coping approaches to stress.

### Sample Size and Sampling Technique

The sample size was calculated using Open-Epi version 2 with consideration of a 5% margin of error, 95% confidence interval, a 50% prevalence of perceived stress associated with the COVID-19 outbreak, and a 1,528 population size. Adding a non-response rate of 10%, the final sample size was 337 students. The participants were recruited by a non-probability sampling technique (a combination of purposive and snowball sampling techniques).

### Data Collection Instrument and Process

The surveillance questionnaire was developed on Google Forms following the review of different kinds of literature. The questionnaire has five sections; sociodemographic characteristics, COVID-19-related experience, WHO precautionary measures, perceived stress scale (PSS-10), and Brief-COPE scale (see Supplementary Data for the collection format).

The perceived stress scale (PSS)-10 (Program, 2020) is the most widely used and validated psychological instrument for measuring the perception of stress and it has also been validated in Ethiopian University students (Manzar et al., 2019). The PSS-10 includes direct queries about current levels of experienced stress. The questions in the PSS ask about the feelings and thoughts of the last month. In each case, respondents are asked how often they felt a certain way. For instance, “In the last month, how often have you been upset because of something that happened unexpectedly?,” “In the last month, how often have you felt that you were unable to control the important things in your life?,” etc.

Each item provides five response options: (i.e., 0 = never, 1 = almost never, 2 = sometimes, 3 = fairly often, 4 = very often). PSS scores were obtained by reversing responses (0 = 4, 1 = 3, 2 = 2, 3 = 1, & 4 = 0) to the four positively stated items (4, 5, 7, and 8). Total scores were obtained by summing all the scale items with a total score range between 0 and 40. In this study, PSS-10 scores equal to or higher than 25 were deemed as high perceived stress, while scores <25 were considered as low perceived stress associated with COVID-19 (Pedrozo-Pupo et al., 2020).

#### Brief-COPE Strategy Scale

The Brief-COPE (NovoPsych, 2020) scale is a 28-item self-report questionnaire designed to measure effective and ineffective ways to cope with a stressful life event. To list some, “I've been turning to work or other activities to take my mind off things,” “I've been concentrating my efforts on doing something about the situation I'm in,” etc. Each question has a 4-point Likert item from “I haven't been doing this at all” to “I've been doing this a lot” (1 = I haven't been doing this at all, 2 = I've been doing this a little bit, 3 = I've been doing this a medium amount, 4 = I've been doing this a lot). The tool has been used in a previous study in Ethiopia (Telake Azale et al., 2018). The term “coping” is defined broadly as an effort used to minimize distress associated with negative life experiences. The coping approaches are broadly categorized as avoidant coping, approach coping, and neither/or, with each category containing subscales.

1. Avoidant coping: characterized by the subscales of denial, substance use, venting, behavioral disengagement, self-distraction, and self-blame. Avoidant coping is associated with poorer physical health among those with medical conditions.
2. Approach coping: characterized by the subscales of active coping, positive reframing, planning, acceptance, seeking emotional support, and seeking informational support. Approach coping is associated with more helpful responses to adversity, including adaptive practical adjustment, better physical health outcomes, and more stable emotional responses.
3. Neither/or: characterized by the subscales of humor and religious ways of coping with stress.

First, each class's representative phone number was accessed from respective departments and we called to them to justify the purpose of the study. Following this, the survey link was then forwarded by Telegram to the representative of each class using their phone numbers. Thereafter, the representative of each class shared the link with the common Telegram address of their class, using a purposive sampling technique. Finally, students who saw the link in the common Telegram address shared the link further to their classmates via Facebook and WhatsApp, using the snowball sampling technique.

In our study, using the Cronbach alpha, the Brief-COPE-28 item (Cronbach alpha equal to 0.844) and the PSS-10 item (Cronbach alpha equal to 0.741) had high and good internal consistencies, respectively. The avoidant coping, approach coping, and neither/or broad categories of coping approaches had good (Cronbach alpha equal to 0.777), high (Cronbach alpha equal to 0.812), and poor (Cronbach alpha equal to 0.455) internal consistencies, respectively.

### Data Quality Assurance

The questionnaires were translated from English to two dominant local languages (Amharic and Afan Oromo) and back-translated into English by an independent person to assure its consistency. The tool was pre-tested on private college health science students, before starting the actual data collection and then any necessary adjustment was done. The data were compiled, coded, and checked for consistency before analysis.

### Data Analysis

The CSV formatted data from Google Forms were exported to SPSS version 22.0 for analysis. A continuous data normality test was conducted using Shapiro-Wilk's *W*-test. For this purpose, a level of significance of 0.05 was used. Parametric data were reported with mean and standard deviation, whereas non-parametric data were reported with the median. Frequency and percentage were computed for categorical variables and a chi-square test was conducted to check for cell adequacy. Bivariate logistic regression was carried out to investigate the associations between the perceived stress level and independent variables/coping strategies. Then, a backward, stepwise multivariate logistic regression [reported with adjusted odds ratios (AOR) with 95% confidence intervals (95% CI)] was performed including all explanatory variables with a *p* < 0.25 on bivariate logistic regression to evaluate factors independently associated with high perceived stress level. All *p*-values calculated were two-sided, and the statistical significance threshold was <0.05.

## Results

### Baseline Characteristics

A total of 337 health science students were involved in the study. The overall mean (±SD) age of the participants was 22.88 (±1.78) years, and the participants were comparable in terms of sex (male = 51.6% vs. female = 48.4%). Nearly three-quarters (73.3%) of the participants reported urban residence (Table 1).

**Table 1:** Socio-demographic characteristics of the study participants.

**Variables**		**Frequency (%)**
Age (mean ± SD), years		22.88 ± 1.78
Sex	Male	174 (51.6)
	Female	163 (48.4)
Residency	Urban	247 (73.3)
	Rural	90 (26.7)
Live with your parents or family	Yes	323 (95.8)
	No	14 (4.2)
Family size	1–4	42 (12.5)
	5–7	64 (19.0)
	8–9	145 (43.0)
	>9	86 (25.5)
Marital status	Single	297 (88.1)
	Married	39 (11.6)
	Divorced	1 (0.3)
Study field	Anesthesia	22 (6.5)
	Generic nursing	91 (27.0)
	Generic midwifery	55 (16.3)
	Environmental health	21 (6.2)
	Operational theater nursing	8 (2.4)
	Neonatal nursing	55 (17.2)
	Pharmacy	71 (21.1)
	Laboratory technology	11 (3.3)
Academic year	1st year	4 (1.2)
	2nd year	146 (43.3)
	3rd year	72 (21.4)
	4th year	61 (18.1)
	5th year	28 (8.3)
	6th year	26 (7.7)
Family/parents monthly estimated income (ETB) (median)		5,000

*SD, standard deviation; ETB, Ethiopian Birr*.

### COVID-19 Related Experiences

A total of 160 (47.5%) students reported predominant use of social media and mass media as the primary source of COVID-19-related information. Most of the students, 298 (88.4%), believe that COVID-19 will have negative consequences on their education. Furthermore, the majority of the participants, 188 (55.8%), think that their family members are at risk of contracting COVID-19, mainly due to work exposure. Above half of the students, 176 (52.2%), reported frustration from the isolation and quarantine measures imposed by the government. Confusion from the inconsistency of health/government authorities' strategies with the scientific recommendations was reported by 199 (59.1%). Regarding income after the COVID-19 outbreak, 213 (63.2%) participants reported a decrease in household income (Table 2).

**Table 2:** COVID-19-related experience among the participants.

**Variables**		**Frequency (%)**
The primary source of COVID-19-related information	Social media and mass media	160 (47.5)
	Social media	110 (32.6)
	Mass media (TV, radio)	77 (22.8)
	Community (family, friends.)	20 (5.9)
Judgment toward accessed information on COVID-19	Correct and balanced	184 (54.6)
	Too alarming	107 (31.8)
	Hides reality so as not to scare	46 (13.6)
Stressed by the daily COVID-19 cases/deaths reported in Ethiopia	Yes	260 (77.2)
	No	77 (22.8)
Stressed by the daily COVID-19 cases/deaths reported worldwide	Yes	278 (82.5)
	No	59 (17.5)
Talk on COVID-19-related updates with parents/friends	Yes	310 (92.0)
	No	27 (8.0)
Frequency of online talk/chat with close friends since the closure of the University	Every day or almost every day	111 (32.9)
	Several times a week	104 (30.9)
	About once a week	59 (17.5)
	Rarely	63 (18.7)
Perception of COVID-19 has negative consequences in education	Yes	298 (88.4)
	No	18 (5.3)
	I don't know	21 (6.3)
Perception of COVID-19-related changes in friendship behavior	Yes	240 (71.2)
	No	69 (20.5)
	I don't know	28 (8.3)
Perception of themselves or family members being at risk of getting sick from the coronavirus	Yes	188 (55.8)
	No	149 (44.2)
Reason for perception of themselves or family members being at risk of getting sick from the coronavirus	Due to existing medical condition	76 (33.3)
	Due to work exposure	115 (50.4)
	Less execution of measures	32 (14.0)
	I don't know	40 (17.5)
Perception of the restrictions (i.e., social distancing, facemask usage) that have been recommended by the local and national government	I think the restrictions are not strict enough	207 (61.4)
	I think the restrictions are too strict	39 (11.6)
	I think the restrictions are appropriate	91 (27.0)
Frustration by the isolation and quarantine measures taken by the government	Yes	176 (52.2)
	No	161 (47.8)
Presence of confusion due to the inconsistent strategies developed by the health/government authorities given the scientific recommendations	Yes	199 (59.1)
	No	138 (40.9)
Presence of change in family behavior following the outbreak of COVID-19	Yes	240 (71.2)
	No	97 (28.8)
Presence of family lifestyle change following the COVID-19 outbreak	Yes	283 (84.0)
	No	54 (16.0)
Directions of family lifestyle change following the COVID-19 outbreak, if any	Positively	218 (76.0)
	Negatively	69 (24.0)
Changes in household income following the COVID-19 outbreak	Decreased	213 (63.2)
	Increased	38 (11.3)
	Not changed	86 (25.5)

### Measures Taken for the Prevention of COVID-19

In this study, a significantly higher proportion of the participants reported practicing all aspects of the COVID-19 infection prevention modalities as per the WHO recommendations (Table 3).

**Table 3:** Measures taken for the prevention of COVID-19 among the participants.

**Variables**		**Frequency (%)**
1. Wash your hands regularly using soap and water for at least 20 s	Yes	301 (89.3)
	No	36 (10.7)
2. Avoid touching your eyes, nose, and mouth with your hand/fingers	Yes	286 (84.9)
	No	51 (15.1)
3. Covering mouth and nose when coughing or sneezing, and washing your hands after	Yes	319 (94.7)
	No	18 (5.3)
4. Avoid close contact with anyone who is sick, especially those with flu or cold symptoms such as fever, cough, or sneezing	Yes	313 (92.9)
	No	24 (7.1)
5. Clean and disinfect frequently touched objects and surfaces	Yes	271 (80.4)
	No	66 (19.6)
6. Stay at home, except to get emergency needs	Yes	272 (80.7)
	No	65 (19.3)
7. Avoid shaking hands with others	Yes	316 (93.8)
	No	21 (6.2)
8. Wearing a face mask while going out of your home	Yes	314 (93.2)
	No	23 (6.8)
9. Avoiding crowds	Yes	318 (93.5)
	No	22 (6.5)

### Perceived Stress Scale

The overall mean (±SD) PSS score was 22.16 (±1.41). The item, “in the last month, how often have you been angered because of things that were outside of your control?,” achieved the highest PPS score with a mean (±SD) value of 2.44 (±1.26), and the item with the lowest PSS score was, “in the last month, how often have you felt that things were going your way?,” with a mean (±SD) PSS score of 2.05 (±1.25) (Table 4). In this study, high perceived stress levels were reported by 121 (35.5%) of the participants, while low perceived stress was reported by 216 (64.5%) of them.

**Table 4:** Perceived stress scale (PSS) among the participants.

**PSS items**	**Average score (mean ± SD)**
1. In the last month, how often have you been upset because of something that happened unexpectedly?	2.16 ± 1.32
2. In the last month, how often have you felt that you were unable to control the important things in your life?	2.21 ± 1.27
3. In the last month, how often have you felt nervous and “stressed”?	2.25 ± 1.24
4. In the last month, how often have you felt confident about your ability to handle your problems?	2.37 ± 1.18
5. In the last month, how often have you felt that things were going your way?	2.05 ± 1.25
6. In the last month, how often have you found that you could not cope with all the things that you had to do?	2.10 ± 1.25
7. In the last month, how often have you been able to control irritations in your life?	2.27 ± 1.15
8. In the last month, how often have you felt that you were on top of things?	2.12 ± 1.25
9. In the last month, how often have you been angered because of things that were outside of your control?	2.44 ± 1.26
10. In the last month, how often have you felt difficulties were piling up so high that you could not overcome them?	2.20 ± 1.22
Total PSS score (M ± SD)	22.17 ± 6.82

*PSS, perceived stress scale; SD, standard deviation*.

### Coping Strategies

The students have reported various means of coping with COVID-19-related stress experiences. Based on the Brief-COPE strategy, the mean (±SD) of the total coping score was 72.34 (±12.31). Of the broad categories of coping approaches, approach coping achieved the highest mean (±SD) score, 33.76 (±6.64). Among subscales of coping strategies, religion achieved the highest mean (±SD) score accounting for 6.35 (±1.60), while substance use was the least stress coping strategy reported with a score of 3.41 (±1.91) (Table 5).

**Table 5:** Brief coping scale among participants.

**Coping styles**		**Coping score (mean ± SD)**
Avoidant coping	Self-distraction, items 1 and 19	5.87 ± 1.42
	Denial, items 3 and 8	4.45 ± 1.86
	Substance use, items 4 and 11	3.41 ± 1.94
	Behavioral disengagement, items 6 and 16	4.80 ± 1.74
	Venting, items 9 and 21	4.85 ± 1.55
	Self-blame, items 13 and 26	4.40 ± 1.83
	Total avoidant coping score	27.78 ± 6.72
Approach coping	Active coping, items 2 and 7	5.80 ± 1.61
	Emotional support, items 5 and 15	5.10 ± 1.62
	Use of informational support, items 10 and 23	5.35 ± 1.71
	Positive reframing, items 12 and 17	5.63 ± 1.51
	Planning, items 14 and 25	5.91 ± 1.53
	Acceptance, items 20 and 24	5.96 ± 1.52
	Total approach coping	33.76 ± 6.64
Neither/or	Humor, items 18 and 28	4.45 ± 1.78
	Religion, items 22 and 27	6.35 ± 1.60
	Total	10.80 ± 2.43
Total coping score	72.34 ± 12.31	

*SD, standard deviation*.

### Factors Associated With the Perceived Stress Level

In bivariate logistic regression, 29 variables achieved a *p* < 0.25 and were recruited for multivariate analysis. Of the 29 candidate variables, family size of 8–9 (*p* = 0.02), personal perception of being stressed by the daily number of COVID-19 cases/deaths in Ethiopia (*p* < 0.001), perception of COVID-19-related changes in friendship behavior (*p* = 0.02), perception of family members at risk of getting sick from the coronavirus (*p* < 0.001), frustration due to the isolation and quarantine measures taken by the government (*p* = 0.01), presence of confusion due to the inconsistent strategies developed by health/government authorities in view of scientific recommendations (*p* < 0.001), presence of a change in family behavior following the outbreak of COVID-19 (*p* < 0.001), presence of family lifestyle change following the COVID-19 outbreak (*p* =0.025), inconsistency of the household income following the COVID-19 outbreak (decreased, *p* = 0.002 and increased, *p* = 0.004), self-distraction, items 1 and 19 (*p* = 0.19), denial, items 3 and 8 (*p* < 0.01), substance use, items 4 and 11 (*p* = 0.01), behavioral disengagement, items 6 and 16 (p < 0.01), venting, items 9 and 21 (*p* < 0.01), self-blame, items 13 and 26 (*p* < 0.01), active coping, items 2 and 7 (*p* = 0.03), emotional support, items 5 and 15 (*p* = 0.04), use of informational support, items 10 and 23 (*p* < 0.01), positive reframing, items 12 and 17 (*p* < 0.01), planning, items 14 and 25 (*p* < 0.01), and religion, items 22 and 27 (*p* < 0.01) were significantly associated with perceived stress level on the bivariate logistic regression analysis.

On multivariate logistic regression analysis, personal perception of being stressed by the daily number of COVID-19 cases/deaths in Ethiopia [AOR = 4.61, 95% CI (1.57–13.50), *P* < 0.01], rare online talk/chat with friends [AOR = 4.07, 95% CI (1.77–9.35), *p* = 0.01], presence of confusion due to the inconsistent strategies developed by health/government authorities in view of the scientific recommendations [AOR = 2.22, 95% CI (1.19–4.14), *p* = 0.01], perception of self/family members being at risk of getting sick [AOR = 0.53, 95%CI (0.29–0.95), *p* = 0.03], and decreased household income following the COVID-19 pandemic [AOR = 3.92, 95% CI (1.36–11.33), *p* = 0.01] were independently associated with perceived stress level. Among subscales of coping strategy, denial, items 3 and 8 [AOR = 1.34, 95% CI (1.11–1.62), *p* < 0.01], self-blame, items 13 and 26 [AOR = 1.23, 95% CI (1.02–1.49), *p* = 0.02], planning, items 14 and 25 [AOR = 1.28, 95% CI (1.04–1.58), *p* = 0.01], and religion, items 22 and 27 [AOR = 1.41, 95% CI (1.14–1.75), *p* < 0.01] means of coping were associated with perceived stress (Table 6).

**Table 6:** Bivariate and multivariate analysis to identify factors associated with perceived stress level.

**Variables**		**Perceived stress level**		**COR [95%CI]**	***p*-value**	**AOR [95%CI]**	***p*-value**
		**Low (*n* = 216)**	**High (*n* = 121)**				
Sex	Female	110 (32.6)	53 (15.7)	0.75 [0.48–1.17]	0.21	–	
	Male	106 (31.5)	68 (20.2)	1			
Age (years)		22.9 ± 2.81	22.7 ± 2.73	0.97 [0.90–1.06]	0.59		
Residency	Rural	52 (15.4)	38 (11.3)	1.44 [0.88–2.36]	0.14	0.55 [0.28–1.08]	0.08
	Urban	164 (48.7)	83 (24.6)	1			
Family size	>9	59 (17.5)	27 (8.0)	0.45 [0.21–0.97]	0.04	–	
	8–9	101 (30.0)	44 (13.1)	0.43 [0.21–0.87]	0.02	–	
	5–7	35 (10.4)	29 (8.6)	0.82 [0.38–1.80]	0.63	–	
	1–4	21 (6.2)	21 (6.2)	1			
Stressed by the no. of COVID-19 cases/deaths in Ethiopia	Yes	155 (46.0)	105 (31.2)	0.38 [0.21–0.70]	<0.01	4.61 [1.57–13.50]	<0.01
	No	61 (18.1)	16(4.7)				
Stressed by the no. of COVID-19 cases/deaths worldwide	Yes	174 (51.6)	104 (30.9)	0.67 [0.36–1.25]	0.21	3.01 [0.94–9.61]	0.06
	No	42 (12.5)	17 (5.0)	1		1	
Frequency of online talk/chat with friends	Rarely	34 (10.1)	29 (8.6)	1.51 [0.80–2.84]	0.19	4.07	0.01
	About once a week	36 (10.7)	23 (6.8)	1.13 [0.59–2.17]	0.70	2.82 [1.19–6.70]	0.01
	Several times a week	75 (22.3)	29 (8.6)	0.68 [0.38–1.22]	0.20	1.23 [0.60–2.51]	0.56
	Every day or almost every day	71 (21.1)	40 (11.9)	1		1	
Perception of COVID-19 has negative consequences in education	Yes	186 (55.2)	112 (33.2)	0.51 [0.18–1.45]	0.21	–	
	I don't know	16 (4.7%)	5 (1.5)	0.47 [0.15–1.47]	0.19	–	
	No	14 (4.2)	4 (1.2)	1			
Perception of COVID-19-related changes friendship behavior	Yes	146 (43.3)	94 (27.9)	0.86 [0.38–1.95]	0.72	–	
	No	52 (15.4)	17 (5.0)	0.50 [0.27–0.93]	0.02	–	
	I don't know	18 (5.3)	10 (3.0)	1			
Perception of themselves or family members being at risk of getting sick from the coronavirus	Yes	106 (31.5)	82 (24.3)	0.45 [0.28–0.73]	<0.01	0.53 [0.29–0.95]	0.03
	No	110 (32.6)	39 (11.6)	1			
Frustration by the isolation and quarantine measures taken by the government	Yes	102 (30.3)	74 (22.0)	0.56 [0.36–0.89]	0.01	–	
	No	114 (33.8)	47 (13.9)	1			
Presence of confusion due to the inconsistent strategies developed by the health/government authorities given the scientific recommendations	Yes	112 (33.2)	87 (25.8)	2.37 [1.47–3.83]	<0.01	2.22 [1.19–4.14]	0.01
	No	104 (30.9)	34 (10.1)	1		1	
Presence of change in family behavior following the outbreak of COVID-19	Yes	143 (42.4)	97 (28.8)	0.48 [0.28–0.82]	<0.01	–	
	No	73 (21.7)	24 (7.1)	1			
Presence of family lifestyle change following the COVID-19 outbreak	Yes	174 (51.6)	109 (32.3)	0.45 [0.23–0.90]	0.02	–	
	No	42 (12.5)	12 (3.6)	1			
Changes in household income following the COVID-19 outbreak	Decreased	128 (38.0)	85 (25.2)	2.50 [1.39–4.51]	<0.01	3.92 [1.36–11.33]	0.01
	Increased	20 (5.9)	18 (5.3)	3.40 [1.49–7.73]	<0.01	1.35 [0.66–2.74]	0.40
	Not changed	68 (20.2)	18 (5.3)	1		1	
Practice of avoiding close contact with anyone who is sick	Yes	197 (58.5)	116 [34.4]	2.23 [0.81–6.15]	0.11	–	
	No	19 (5.6)	5 (1.5)	1			
Practice of avoiding shaking hands with others	Yes	199 (59.1)	117 (34.7)	0.40 [0.13–1.21]	0.10	0.24 [0.06–1.02]	0.05
	No	17 (5.0)	4 (1.2)	1		–	
Avoidant coping	Self-distraction, items 1 and 19^*^	5.80 ± 1.429	6.01 ± 1.417	1.11 [0.94–1.30]	0.19	–	
	Denial, items 3 and 8^*^	4.04 ± 1.690	5.18 ± 1.932	1.41 [1.24–1.61]	<0.01	1.34 [1.11–1.62]	<0.01
	Substance use, items 4 and 11^*^	3.21 ± 1.749	3.78 ± 2.223	1.15 [1.03–1.29]	0.01	–	
	Behavioral disengagement, items 6 and 16^*^	4.52 ± 1.773	5.30 ± 1.590	1.30 [1.13–1.49]	<0.01	–	
	Venting, items 9 and 21^*^	4.51 ± 1.494	5.46 ± 1.478	1.53 [1.30–1.80]	<0.01	1.22 [0.98–1.51]	0.06
	Self-blame, items 13 and 26^*^	4.00 ± 1.719	5.10 ± 1.828	1.40 [1.23–1.60]	<0.01	1.23 [1.02–1.49]	0.02
Approach coping	Active coping, items 2 and 7^*^	5.66 ± 1.646	6.05 ± 1.527	1.16 [1.01–1.34]	0.03	–	
	Emotional support, items 5 and 15^*^	4.96 ± 1.698	5.34 ± 1.464	1.15 [1.00–1.32]	0.04	–	
	Use of informational support, items 10 and 23^*^	5.07 ± 1.688	5.84 ± 1.658	1.31 [1.14–1.51]	<0.01	–	
	Positive reframing, items 12 and 17^*^	5.39 ± 1.566	6.06 ± 1.318	1.35 [1.16–1.59]	<0.01	–	
	Planning, items 14 and 25^*^	5.68 ± 1.630	6.33 ± 1.261	1.33 [1.14–1.56]	<0.01	1.28 [1.04–1.58]	0.01
	Acceptance, items 20 and 24^*^	5.89 ± 1.598	6.09 ± 1.384	1.09 [0.94–1.26]	0.24	–	
Neither/or coping	Humor, items 18 and 28^*^	4.36 ± 1.770	4.62 ± 1.795	1.08 [0.95–1.23]	0.19	0.84 [0.70–1.00]	0.06
	Religion, items 22 and 27^*^	6.13 ± 1.717	6.75 ± 1.306	1.30 [1.11–1.51]	<0.01	1.41 [1.14–1.75]	<0.01

*SD, standard deviation*;

* *reported in mean ± standard deviation*.

## Discussion

This cross-sectional surveillance study was undertaken involving 337 undergraduate health science students to assess the perceived stress level and coping strategies amid the COVID-19 pandemic. More than one-third (35.9%) of participants experienced high perceived stress. The overall mean PSS score was 22.17 (±6.82), whereas the total mean of the stress coping score was 72.34 (±12.31).

The prevalence of high perceived stress was relatively higher in the current study than in other previous studies. In Colombia, Pedrozo-Pupo et al. (2020) found that 14.3% of the participants reported high perceived distress during the COVID-19 pandemic. In another study, Sheroun et al. (2020) reported that 13.3% of Bachelor science of nursing students suffered from high perceived stress. This difference might be explained by the difference in the study population; unlike the current study, the Pedrozo et al. study recruited professors, students, and health professionals, while, Sheroun et al. enrolled only Bachelor science nursing students. Which might reflect the significant difference in perceived stress level across different populations and, additionally, it may show cross-country discrepancies of the PSS amid the COVID-19 pandemic.

During a crisis, such as the outbreak of COVID-19, effective coping strategies play a vital role in managing distress and developing resilience. The type of stress coping employed in such circumstances is believed to impact health differently resulting in either poor or better health outcomes (NovoPsych, 2020; Polizzi et al., 2020). In our study, among subscales of coping, religion as a means of coping with stress achieved the highest mean score followed by planning and acceptance. On the contrary, substance use was the lowest means reported for coping with stress. Similarly, a study on medical students from Qatar reported religion, planning, and acceptance as the three most frequent means of coping with stress. Moreover, the Qatar study corroborates our finding of substance use as the lowest used means of coping with stress (Slah Eddine and Adawi, 2020). Overall, of a broad categories of coping, our study participants reported approach coping as a major means of coping with stress. This implies that the majority of the students were practicing effective means of coping with stress, which is associated with better physical health outcomes (NovoPsych, 2020).

Students who perceived being stressed by the daily number of COVID-19 cases/deaths in Ethiopia were four times more likely to have a high perceived stress level. This might be explained by an increment in alarming information that led to a drastic increase in stress-related conditions and mental health issues (Fernandes, 2020; Vinkers et al., 2020). Consistent with a study report from Colombia (Pedrozo-Pupo et al., 2020), our study also identified an association between confusion due to the inconsistent strategies developed by the health/government authorities in view of scientific recommendations and high perceived stress level. Inconsistent recommendations and messaging by health/government authorities leads to confusion or omitting steps or making other errors which can result in an increased risk of contamination and infection, which ultimately distress the individual (Peters et al., 2020). There is evidence that social media exposure is associated with psychological distress among college students (Hong et al., 2020) and similarly, poor social interaction (less sharing of ideas with friends, families, and community) influenced students' perceptions of stress (Stoliker and Lafreniere, 2015). In the current study, students who rarely talk/chat with their friends online were at a more than four-fold higher risk of having high perceived stress compared with those who talk/chat every day in the current pandemic. In the present study, decreased household income following the COVID-19 outbreak increased the risk of high perceived stress. This finding is consistent with a study report from Italy (Flesia et al., 2020). The emergency of the COVID-19 pandemic has caused a multitude of crises, one of which is economic (Bank, 2020). Decreased household income results in the inability to execute daily demands; this challenge will increase personal stress level.

Among subscales of coping strategies reported by the students, denial, self-blame, planning, and religion significantly increased the risk for high perceived stress. Denial and self-blame are part of the avoidance coping strategies of trying to avoid stressors instead of dealing with them. They are maladaptive (or unhealthy) ways of coping with stress because they often exacerbate stress without helping a person deal with the things that are causing them stress (Dijkstra and Homan, 2016). The increased risk of high perceived stress by using religion and planning as stress coping strategies could be an unfortunate finding or could be related to the unprecedented impact of COVID-19 on activities like religious practices and planning.

The present study has some limitations. Selection bias due to the sampling techniques employed dictates the cautious interpretation of the findings. Additionally, the authors adopted an existing scale to measure perceived stress and coping strategies associated with COVID-19. Furthermore, the study only enrolled health science students from a single institution, which might limit inference to health science students throughout Ethiopia as well as students of other subjects in the same institution.

## Conclusion

More than one-third of the participants had a high level of perceived stress, and the majority of them were practicing effective means of coping with stress. Personal perception of being stressed by the daily number of COVID-19 cases/deaths in Ethiopia, rare online talk/chat with friends, presence of confusion due to the inconsistent strategies developed by the health/government authorities in view of scientific recommendations, and decreased household income following the COVID-19 pandemic were the predicting factors of high perceived stress level. Among the subscales of coping strategies, denial, self-blame, planning, and religious means of coping with stress were associated with high perceived stress. The authors recommend that the hosting University in collaboration with the concerned bodies develop innovative strategies to improve the psychological well-being of the students in parallel with class courses using online platforms as well as expanding the existing student counseling facilities. The health/government authorities have to develop consistent strategies in view of the scientific recommendations to contain the pandemic. Besides, longitudinal studies have to be conducted to assess the long-term psychological/psychiatric impact of the COVID-19 pandemic as well as the cognitive and academic performance impact of it. Moreover, research with large sample sizes and identifying the sources of stress among the students are warranted.

## Data Availability Statement

The original data presented in the study are included in the article/Supplementary Material, further inquiries can be directed to the corresponding author/s.

## Ethics Statement

The studies involving human participants were reviewed and approved by Institutional review board (IRB) of Jimma University, Ethiopia. The patients/participants provided their written informed consent to participate in this study.

## Author Contributions

MA, BT, SA, and GM conceptualized and designed the study. MA and BT analyzed the data, interpreted the findings, and wrote the manuscript. All authors read and approved the final manuscript.

## Conflict of Interest

The authors declare that the research was conducted in the absence of any commercial or financial relationships that could be construed as a potential conflict of interest.

## Abbreviations

AOR: adjusted odds ratios
COVID-19: coronavirus infection disease-2019
ETB: Ethiopian Birr
M: mean
PSS: perceived stress scale
SD: standard deviation
SPSS: Statistical Package for Social Science.
