# Supplementary material for: Perceived Stress and Coping Strategies Among Undergraduate Health Science Students of Jimma University Amid the COVID-19 Outbreak: Online Cross-Sectional Survey
Source: Front Psychol. 2021 Mar 30;12:639955. doi: 10.3389/fpsyg.2021.639955 (PMC8042268; doi:10.3389/fpsyg.2021.639955)
Supplement: Supplementary file 1 [file Data_Sheet_1.docx]

**APPENDIX 1: Data abstraction format**

Jimma University, Institute of Health Science

Perceived stress and coping strategies among undergraduate health science students of Jimma University in the era of COVID-19 outbreaks: online surveying.

The study aimed to assess perceived stress and coping strategies among undergraduate Anesthesia, Environmental health, pharmacy, Generic nursing, Generic midwifery, neonatal nursing, operational theatre nursing, and Laboratory technology students at Jimma University in the era of covid-19 outbreaks.
The assessment tool was developed based on the current pandemic and it contains questions to be responded based on your feelings, experiences, and thoughts about the COVID-19 pandemic.

The tool contains five sections:
1. Demographic characteristics
2. COVID-19 related experience
3. Measures taking for prevention of COVID-19
4. Perceived Stress assessment
5. Brief of Coping strategies

Investigators:
Mengist Awoke, Email: [mengist93@gmail.com](mailto:mengist93@gmail.com), Phone.no:+251913567977
Behailu Terefe, Email: [terefebh@gmail.com](mailto:terefebh@gmail.com), Phone.no:+251920194883
Girma Mamo, Email: [girma.mamo2004@gmail.com](mailto:girma.mamo2004@gmail.com), Phone.no:+251917805608
Samuel Abdu, Email: [samuelabdu2004@gmail.com](mailto:samuelabdu2004@gmail.com), Phone.no:+251906573156

**Demographic characteristics**

1. Age (years) Your answer ______
2. Sex
3. Female
4. Male
5. Region/City of residency
6. Addis Abeba
7. Afar
8. Amhara
9. Benishangul Gumuz
10. Diredewa
11. Gambella
12. Hareri
13. Oromia
14. Somalia
15. Southern Nation, Nationalities and People Region (SNNPR)
16. Sidama Region
17. Tigray
18. Residency
19. Urban
20. Rural
21. Do you live with your parents or Family?
22. Yes
23. No
24. How much is your family size? ______
25. Field of Study
26. Anesthesia
27. Environmental health
28. Generic Nursing
29. Generic Midwifery
30. Operational Theatre Nursing
31. Neonatal Nursing
32. Pharmacy
33. Laboratory technology
34. Study year
35. 1st year
36. 2nd year
37. 3rd year
38. 4th year
39. 6th year
40. Marital Status
41. Single
42. Married
43. Divorced
44. Widowed

10. Family or your monthly estimated income______ETB

**Covid-19 related experiences**

The following questions are constructed based on experiences probably encountered in your life during the epidemic. Please read each item carefully and then select the corresponding option in each question that matches your experience.

1. What is your primary source of COVID-19 related information?
2. Social media
3. Mass media (TV, Radio)
4. Social media and mass media
5. Community (family, friends...)
6. What is your judgment towards accessed information on COVID-19?
7. Too much alarming
8. Hide reality not to scare
9. Correct and balanced
10. are you stressed by the daily number of COVID-19 cases/deaths reported in Ethiopia?
11. Yes
12. No
13. are you stressed by the daily number of COVID-19 cases/deaths reported worldwide?
14. Yes
15. No
16. Do you talk on COVID-19 related updates with parents/friends?
17. Yes
18. No
19. Since the university has closed, how often do you talk/chat with friends online (including on your cell phone, on social media, or through online gaming)?
20. Every day or almost every day
21. Several times a week
22. About once a week
23. Rarely
24. Do you ever think COVID-19 will have negative consequences in your education?
25. Yes
26. No
27. I don't know
28. Do you think COVID-19 changes your friendship behavior?
29. Yes
30. No
31. I don't know
32. Do you think you or your family members are at risk of getting sick from the coronavirus?
33. Yes
34. No
35. If your response is yes to the above question, why (you can tick more than one option)?
36. Due to existing medical condition
37. Due to work exposure
38. Less execution of measures
39. I don’t know
40. How do you see the restrictions (i.e. social distancing, facemask usage) that have been recommended by your local and national government?
41. I think the restrictions are not strict enough
42. I think the restrictions are too strict
43. I think the restrictions are Appropriate
44. Are you frustrated by isolation and quarantine measures taken by the government?
45. Yes
46. No
47. Have you ever confused by inconsistent strategies developed by health/Government authorities in view of scientific recommendations?
48. Yes
49. No
50. Is there any change in family behavior following the outbreak of COVID-19?
51. Yes
52. No
53. Are you observing family lifestyle change following the COVID-19 outbreak?
54. Yes
55. No
56. If your response is yes for the above question, in what way?
57. Positively
58. Negatively
59. Rate changes in household employment or income following COVID-19 pandemic
60. Decreased
61. Increased
62. Not changed

**Measures taking for prevention of COVID-19**

Answer the following question based on your actual actions taking behavior to prevent COVID-19 infection

1. Wash your hands regularly using soap and water for at least 20 seconds
2. Yes
3. No
4. Avoid touching your eyes, nose, and mouth with your hand/fingers.
5. Yes
6. No
7. Covering mouth and nose when coughing or sneezing, and washing your hands after.
8. Yes
9. No
10. Avoid close contact with anyone who is sick, especially those with flu or cold symptoms such as fever, cough, or sneezing.
11. Yes
12. No
13. Clean and disinfect frequently touched objects and surfaces.
14. Yes
15. No
16. Stay at home, except to get emergency needs.
17. Yes
18. No
19. Avoid shaking hands with others.
20. Yes
21. No
22. Wearing facemask while going out of home
23. Yes
24. No
25. Avoid large gatherings
26. Yes
27. No

**Perceived Stress Scale (due to COVID-19)**

Please answer the next 10 questions in accordance with your feelings or thoughts following the COVID-19 pandemic in the past one month.

**0 = Never, 1 = Almost Never, 2 = Sometimes, 3 = Fairly Often, 4 = Very Often**

1. In the last month, how often have you been upset because of something that happened unexpectedly?

2. In the last month, how often have you felt that you were unable to control the important?

3. In the last month, how often have you felt nervous and “stressed”?

4. In the last month, how often have you felt confident about your ability to handle your personal problems?

5. In the last month, how often have you felt that things were going your way?

6. In the last month, how often have you found that you could not cope with all the things that you had to do?

7. In the last month, how often have you been able to control irritations in your life?

8. In the last month, how often have you felt that you were on top of things?

9. In the last month, how often have you been angered because of things that were outside of your control?

10. In the last month, how often have you felt difficulties were piling up so high that you could not overcome them?

**Brief of coping strategies**

The following lists the attitudes and practices that you may take when you are hit by setbacks or encounter difficulties in your life during the epidemic. Please read each item carefully and then select the corresponding option in each question that matches your situation. 1 = I haven't been doing this at all, 2 = I've been doing this a little bit, 3 = I've been doing this a medium amount, 4 = I've been doing this a lot

1. I've been turning to work or other activities to take my mind off things.

2. I've been concentrating my efforts on doing something about the situation I'm in.

3. I've been saying to myself "this isn't real.".

4. I've been using alcohol or other drugs to make myself feel better

5. I've been getting emotional support from others.

6. I've been giving up trying to deal with it.

7. I've been taking action to try to make the situation better.

8. I've been refusing to believe that it has happened.

9. I've been saying things to let my unpleasant feelings escape.

10. I’ve been getting help and advice from other people.

11. I've been using alcohol or other drugs to help me get through it.

12. I've been trying to see it in a different light, to make it seem more positive.

13. I’ve been criticizing myself.

14. I've been trying to come up with a strategy about what to do.

15. I've been getting comfort and understanding from someone.

16. I've been giving up the attempt to cope.

17. I've been looking for something good in what is happening.

18. I've been making jokes about it.

19. I've been doing something to think about it less, such as watching TV, reading, daydreaming, sleeping, and sport activity.

20. I've been accepting the reality of the fact that it has happened

21. I've been expressing my negative feelings.

22. I've been trying to find comfort in my religion or spiritual beliefs.

23. I’ve been trying to get advice or help from other people about what to do.

24. I've been learning to live with it.

25. I've been thinking hard about what steps to take.

26. I’ve been blaming myself for things that happened.

27. I've been praying or meditating.

28. I've been making fun of the situation.
